# Complete genome annotation of the cluster EC *Microbacterium* phage Nicole72 from Pittsburgh, Pennsylvania

**DOI:** 10.1128/mra.00507-25

**Published:** 2025-07-22

**Authors:** Adam J. Ziegler, Christa P. Joby, Hams A. Kamil, Ethan Johnson, Ashlyn L. Ivey, Vanessa A. Fullante, Gautam Ghosh, Laura Leal Martinez, Gabrielle A. Tutelo, Devya Wilson, Christine A. Byrum

**Affiliations:** 1 Department of Biology, College of Charleston, Charleston, South Carolina, USA; Portland State University, Portland, Oregon, USA

**Keywords:** microbacteriophage, annotation, SEA-PHAGES, genome, actinobacteriophage, bacteriophage, virus, cluster EC, siphovirus, Nicole72

## Abstract

Nicole72, a microbacteriophage isolated from a flowerbed in Pittsburgh, Pennsylvania, has a 55,431 bp genome containing 90 predicted protein-coding genes and no transfer RNAs or transfer-messenger RNAs. Nicole72 has siphovirus morphology and is most closely related to the EC cluster microbacteriophage Megan where BLASTn indicates 81.5% identity, 86% query coverage.

## ANNOUNCEMENT

**Fig 1:**
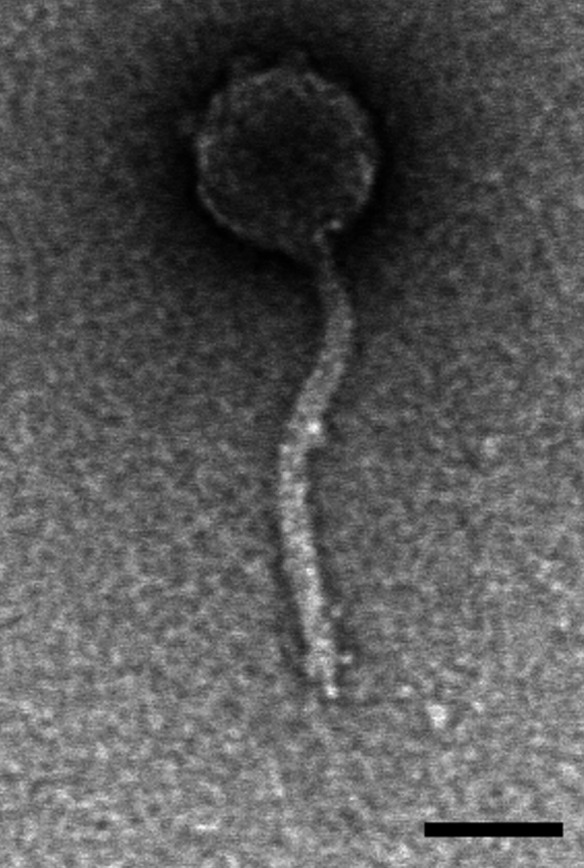
Micrograph of the cluster EC microbacteriophage Nicole72. Viruses were mounted on Formvar-coated copper grids and then negatively stained with 1% uranyl acetate (2). Images were collected using a JEOL 1010 transmission electron microscope at 80 kV. Scale bar equals 50 nm.

The bacteriophage Nicole72 was studied/annotated in a broader effort to better understand actinobacteriophage genomes and evolution (1). It was discovered in 2019 in the soil of an abandoned flowerbed outside William Pitt Union, University of Pennsylvania (40.443649 N, 79.954516 W). After washing this sample (15 mL) in a conical tube filled to 35 mL with 7H9 buffer +1 mM CaCl_2_ (250 rpm, 1–2 hours, 30°C), the supernatant was collected by centrifugation and syringe-filtered (0.22 µm pore). Samples were then inoculated with 0.5 mL *Microbacterium paraoxydans* NWU1, incubated (250 rpm, 3–5 days, 30°C), refiltered, and plated using the double-layer method in 7H9 top agar containing *Microbacterium* host (full protocol in SEA-PHAGES Discovery Guide) (2). Transmission electron microscopy revealed Nicole72 (*n *= 1) has siphovirus morphology (tail length = 170.4 nm; tail width = 10.4 nm; capsid width = 67.8 nm) (Fig. 1).

Using the Promega Wizard DNA cleanup system, DNA was extracted from high-titer lysates (Protocol 7.1, Reference 2) and measured (Nanospec 2000), and a DNA library was prepared per manufacturer instructions using the NEBNext Ultra II DNA library prep kit. The genome was sequenced using an Illumina MiSeq system (v3 reagents) (3), and 32,570 raw reads (150 bp, single-end reads) were assembled into one contig using Newbler v2.9 (4). The genome was edited/finished with Consed v29.0 (3, 5) and this tool was utilized to assess read buildup. AceUtil (http://phagesdb.org/AceUtil) was run to detect sequence discrepancies/low-coverage sites. Finally, the genome was oriented so (i) structural genes appear on the forward strand, and (ii) base 1 is aligned similarly, where possible, with genes in other EC cluster members.

Annotation was performed using the workflow tool PECAAN (6). To predict gene functions, programs used included GLIMMER v3.02 (7), GeneMark v2.5 (8), Starterator v1.2 (https://seaphages.org/media/docs/Starterator_Guide_2016.pdf), ARAGORN v1.2.38 (9), tRNAscan-SE v3.0 (10), and Phamerator Actino_prophage v5 (11). Functional assignments were made using BLASTp v2.13.0+ (12), HHpred (13), and NCBI’s Conserved Domain Database (CDD) (14). Transmembrane domains were detected with TMHMM2 (https://services.healthtech.dtu.dk/service.php?TMHMM-2.0), SOSUI v1.11 (15), and TOPCONS v2 (16). Programs were set to default parameters, and final PECAAN files were transferred to DNA Master v5.22.2 (https://phagesdb.org/DNAMaster).

The Nicole72 genome is 55,431 bp with 69.7% GC and 84× fold coverage. Because even coverage was detected across the genome with no selective accumulation of reads, the genome is circularly permuted (3). Nicole72’s genome contains 90 predicted protein-coding genes (27 with assigned putative functions; 6 membrane proteins) but lacks transfer RNAs/transfer-messenger RNAs. Based on gene content similarity (GCS) scores, Hashim76 is an EC cluster phage (GCS = #phams shared between two genomes ÷ total #phams, where phams are predicted homologous sequences sharing >32.5% amino acid identity in CLUSTALW, BLASTp E-values <10^−50^) (17–19). To check how often each Nicole72 pham corresponded to sequences in other actinobacteriophages, a pull-down menu listing clusters containing the conserved sequence was accessed on Phamerator (11). The Nicole72 genome contains 11 unique phams (orphams) and has 10 phams conserved throughout the EC cluster (>95% of members and only occurring in the EC cluster) (Table 1). GCS scores (19) and BLASTn alignment (12) revealed that the Nicole72 genome most resembles that of microbacteriophage Megan (Genbank MN586020; 78.90% GCS, 85.08% percent identity, 81% coverage). Interestingly, both Megan and Nicole72 are predicted to transcribe preQ0 pathway proteins synthesizing queuosine and archaeosine. Although these sequences are sometimes conserved in other actinobacteriophages, Megan and Nicole72 are the only EC cluster genomes with these (20). Finally, Nicole72 is lytic, since it codes for an endolysin (LysinA) and lacks candidate lysogeny genes.

**TABLE 1:** Characteristics of the Nicole72 bacteriophage

Parameter	Nicole72 data
GenBank accession no.	OR159674
SRA accession no.	SRX21368077
Isolation site	Pittsburgh, PA, USA
Collection site coordinates	40.443649 N; 79.054516 W
Isolation host	*Microbacterium paraoxydans* NWU1
Genome size (bp)	55,431
Approximate shotgun coverage (x)	84
GC content (%)	69.7
No. of predicted protein-coding genes	90
No. of tRNAs	0
No. of tmRNAs	0
Morphotype	Siphovirus morphology
Cluster	EC
Predicted protein-coding genes only found in Nicole72 (orphams)^*a*^	3, 6, 17, 18, 19, 37, 63 (membrane protein), 70 (deoxycytidylate deaminase), 74, 76, and 89
Predicted protein-coding genes (phams) unique to and conserved in at least 95% of EC cluster members^*a*^	24, 29 (tail assembly chaperone), 30, 41 (membrane protein), 43, 48, 50, 69, 72 (membrane protein), and 73 (membrane protein)

^*a*^ Based on data available in Phamerator on 6 May 2025 (11). Functions of the predicted genes are unknown unless named in parentheses.

## Data Availability

The Nicole72 genome is available in Genbank (accession number OR159674.1). Raw reads appear in the Sequence Read Archive (accession number SRX21368077).
